# Noise Modeling of the Overhauser Magnetometer

**DOI:** 10.3390/s25247491

**Published:** 2025-12-09

**Authors:** Xiaorong Gong, Shuang Zhang, Shudong Chen

**Affiliations:** College of Electronic Science and Engineering, Jilin University, Changchun 130012, China; gongxr21@mails.jlu.edu.cn (X.G.); zhangshuang@jlu.edu.cn (S.Z.)

**Keywords:** quantum magnetometer, Overhauser sensor, noise model, noise measurement, sensitivity

## Abstract

The Overhauser magnetometer (OVM) is an electron resonance-enhanced nuclear magnetic resonance (NMR) magnetometer, which significantly enhances the Larmor signal, hence the signal-to-noise ratio (SNR) and sensitivity compared to traditional proton magnetometers (PM). In this paper, we intended to improve SNR and sensitivity only by reducing system noise. For this purpose, an equivalent circuit model of noise is established, and the contributions of sensor and transmission characteristics of the circuit are calculated quantitatively. By sensor parameter optimization, matching resistance, and preamplifier selection to reduce the noise of the system, the root mean square (rms) of system noise is 26.7 mV, which is consistent with the theoretical 23.9 mV. By reducing the noise of the system, the SNR of the Larmor signal can reach 39 dB. The measured results in the natural environment show that the sensitivity of the OVM is 0.0079 nT at 3 s cycling time.

## 1. Introduction

The Overhauser magnetometer (OVM) is a proton precession magnetometer which uses electron–nuclear double resonance to enhance the proton polarization [1]. Its polarization strength is dozens of times higher than that of traditional proton magnetometers [2]. Due to its advantages, such as low power, high sensitivity, high absolute accuracy, excellent portability, and stable performance, the OVM has been widely used in many fields such as geomagnetic field measurement, magnetic target detection, and magnetic device calibration [3,4,5,6].

Sensitivity is a key indicator of the OVM, representing the statistical uncertainty observed during repeated measurements of the same magnetic field [7]. It reflects the instrument’s measurement noise, which is primarily determined by the signal-to-noise ratio (SNR) of the Larmor signal [8]. To improve sensitivity, previous research has mainly focused on two aspects: FID signal processing and hardware optimization.

In terms of signal processing, the primary research focus is on suppressing different types of noise embedded in the free induction decay (FID) signal [9]. Tan et al. [10] proposed an optimal multi-average (OMAM) frequency measurement algorithm that achieved a sensitivity of 0.2 nT/$\sqrt{Hz}$, effectively enhancing frequency precision. Wang et al. [11] introduced a segmented linear regression method to recover FID signals suppressed by saturation, thereby improving signal stability. Luo et al. [12] further proposed an intelligent full-wavenumber fitting algorithm for low-SNR FID signals, achieving 0.067 nT sensitivity and strong robustness against interference. These studies significantly improved frequency extraction accuracy but mainly focused on algorithmic optimization, without analyzing the intrinsic noise sources that fundamentally limit sensitivity.

In terms of hardware, Liu et al. [13] developed an adjustable tuning–matching framework that broadened the sensing coil bandwidth and reduced the noise floor, effectively doubling the SNR and sensitivity under strong interference. Wang et al. [14] designed an integrated dual-mode magnetometer with a sensitivity better than 0.06 nT, demonstrating high precision and system stability. Commercial OVMs, such as those produced by GEM Systems (Canada), have already achieved sensitivities of approximately 0.01 nT.

However, these works primarily addressed circuit filtering optimization and external interference suppression, while the intrinsic noise mechanisms within the sensor and circuit remain insufficiently investigated. By contrast, other weak magnetic field sensing technologies have developed more mature and systematic noise analyses. In magnetoresistive (MR) heads, thermal magnetization fluctuations have been identified as a fundamental white-noise limit that scales inversely with sensor volume [15]. Giant magnetoimpedance (GMI) wires and multilayer thin films have been shown to exhibit intrinsic magnetic noise governed by magnetization dynamics, anisotropy, and damping, with equivalent noise reaching the sub-nT to pT/$\sqrt{Hz}$ range [16,17]. Magnetic tunnel junction (MTJ) magnetometers have further established unified equivalent-noise models that incorporate sensor, front-end electronics, and feedback-coil contributions, with the latter often dominating at low frequencies [18]. These studies highlight the well-developed understanding of intrinsic noise in sensors, whereas intrinsic noise in the OVM remains relatively unexplored.

The study of the inherent noise characteristics of the OVM helps us understand the composition of noise sources and the contributions of various noise sources, and this is important for intrinsic noise suppression and SNR enhancement in order to obtain high sensitivity. For this purpose, an equivalent noise model for OVM is established, and the optimized sensor parameters according to this noise model are used to obtain a high SNR Larmor signal, hence the high sensitivity. First, a comprehensive noise model combined with the sensor and the receiving circuit is proposed to analyze the impact of various types of noise on the SNR of the FID signal. Second, the influence of circuit transmission characteristics on noise is investigated, and the noise transmission characteristics are obtained, and the influence of various electrical parameters of the sensor and circuit on the noise was analyzed. Finally, the correctness of the noise model was verified, and the sensitivity under low instrument background noise was estimated, experimentally. The main contributions of this work are as follows:

(1) A comprehensive noise model is developed to describe the intrinsic noise sources within the OVM, including sensor noise and circuit noise.
(2) The proposed noise model is used to guide the optimization of sensor parameters and circuit design, effectively reducing the total output noise and improving the SNR.
(3) The accuracy of the proposed model and the sensitivity of the optimized OVM are experimentally verified. The results confirm that the developed noise model can accurately reflect system noise behavior and provide theoretical guidance for low-noise design.

Therefore, this study aims to construct a comprehensive quantitative noise model for the OVM, systematically identifying both intrinsic physical noise sources and circuit-induced noise pathways. By resolving the noise contributions of each stage in the analog and validating them through dedicated experiments, the study offers practical design guidance for improving its SNR performance.

## 2. Theory and Design of OVMs

### 2.1. Basic Principle of an OVM

Similar to a traditional proton magnetometer, an OVM measures the frequency of the proton magnetic moment precessing around the external magnetic field to calculate the magnetic field strength. The relationship between Larmor precession frequency *f*_e_ and the external magnetic field strength ***B*** is

(1) $$B=2\pi f_{e}/\gamma_{p}=23.4872f_{e},$$

where $\gamma_{p}$ (2.6751987 × 10^8^ T^−1^·S^−1^) is the gyromagnetic ratio of the hydrogen proton. Usually, the measurement range of the OVM is 20 μT to 120 μT, corresponding to a Larmor frequency range of 851 Hz to 5109 Hz.

Unlike the proton magnetometer which uses a strong prepolarization field (~mT), OVMs employ a more efficient and low-power dynamic nuclear polarization (DNP) method to improve the polarization efficiency of protons. DNP technology is a dual magnetic resonance technology [19,20]. When the RF field acts on a solution rich in free electrons and protons, it will excite the electron spins in the solution to produce electron spin resonance (ESR). Since the electron spins and proton spins in the solution are coupled to each other, when the ESR is saturated, the proton polarization is enhanced through the cross-relaxation process [21,22]. Solomon defined the enhancement strength of proton spin polarization using the dynamic nuclear polarization factor (*DNPF*) [23], which can be expressed as

(2) $$DNPF=\frac{\langle I_{Z}\rangle -I_{0}}{I_{0}}=-\xi sf(\frac{\langle S_{Z}\rangle -S_{0}}{I_{0}}).$$

where $\langle I_{z}\rangle$ and $\langle S_{z}\rangle$ are the spin polarizations of the nuclear spins *I* and electron spins *S* after electron spin resonance, $I_{0}$ and $S_{0}$ are the polarizations at thermal equilibrium. *ξ*, *s*, and *f* are coupling factor, saturation factor, and leakage factor, respectively [24]. Theoretically, the *DNPF* can reach tens of thousands or only a few thousand [25].

**Figure 1:**
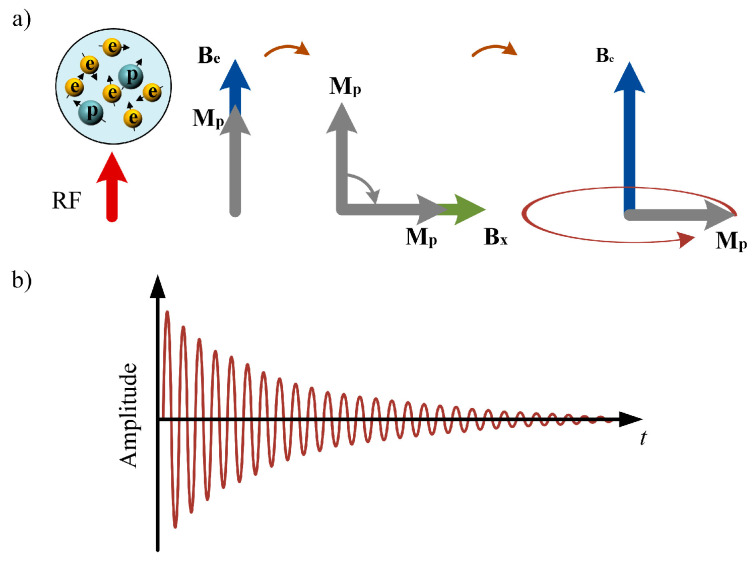
(**a**) Micro-mechanism of OVM sensor. (**b**) FID signal induced by the audio coil.

The operating micro-mechanism of the OVM sensor is shown in Figure 1a. First, the RF signal excites the free radical solution inside the sensor to produce the DNP effect, which enhances the polarization strength of the proton. Then, a 90° DC pulse is used to bias the proton magnetization in the transverse direction. After the pulse disappears, the proton magnetization begins to precess around the Earth’s magnetic field, and the FID signal is induced in the sensor coil (see Figure 1b). The amplitude expression of the FID signal is

(3) $$V(t)=\mu_{0}MAn\omega e^{-t/T_{2}}\sin^{2}\;\theta \sin\;\omega t,$$

where $\mu_{0}$ is the vacuum permeability, *M* is the proton magnetization strength, *A* is the coil cross-sectional area, *n* is the number of turns, ω is the angular frequency of the Larmor signal, $T_{2}$ is the transverse relaxation time, and θ is the angle between the coil axis and the Earth’s magnetic field direction.

### 2.2. OVM Workflow

**Figure 2:**
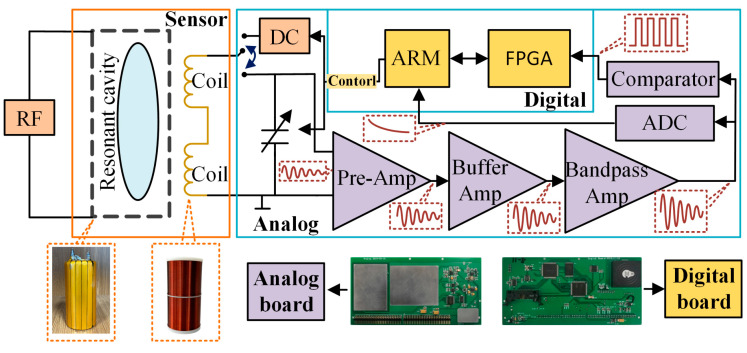
Operating mechanism of OVMs.

According to the above principle, the prototype OVM, as shown in Figure 2, consists of two parts: the sensor and the console.

The sensor of the OVM consists of an audio coil, a capacitor-loaded resonant cavity, and a quartz bottle filled with radical solution. The resonant cavity aimed to generate RF to excite the solution for achieving a DNP effect. High-conductivity metal conductors are used to construct resonant cavities, and gaps are left between the copper foils to avoid eddy current, which can generate unexpected magnetic fields. The audio coil is used to generate a 90° pulse polarization and induce the FID signal, and two solenoid coils are connected in series in opposite directions to prevent external interference.

The console of the OVM consists of two parts: a digital board and an analog board. During the polarization process, the analog board is responsible for generating RF 90° pulse polarization signals. During the receiving process, the LC parallel circuit is used to enhance the FID signal by resonant amplification. The signal is amplified to the voltage level after multi-stage amplification, and the multi-stage amplification is divided into preamplifier, buffer amplifier (LT0613), and bandpass amplifier (LT0621). Subsequently, the FID signal is transmitted to the ARM through the ADC (integrated in the ARM processor) acquisition circuit for signal quality evaluation. The signal is also shaped into a square wave signal by the comparator and sent to the FPGA for frequency measurement. Finally, the ARM converts the frequency value into the geomagnetic field according to (1).

## 3. Analytical Noise Model of OVMs

The audio coil, as an electromagnetic induction sensor, can operate under various conditions, including critical damping in time-domain induction, resonance in frequency-domain induction, and negative feedback in broadband induction. In this work, the OVM operates in the resonant state of frequency-domain induction. By modeling the coil noise under this resonant condition, the coil parameters can be optimized to enhance the system performance and suppress excessive inherent noise.

### 3.1. Noise Model of the Audio Coil and Preamplifier

**Figure 3:**
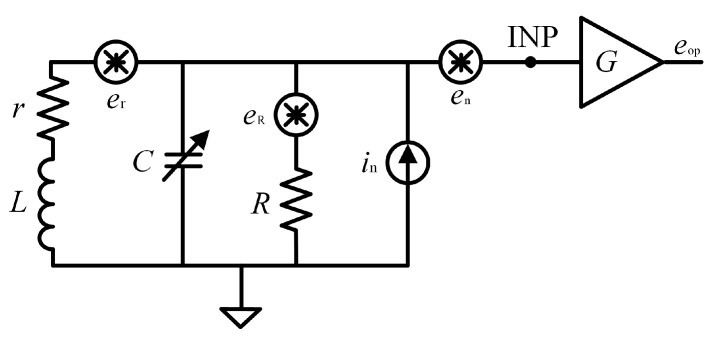
Equivalent circuit of the audio coil and preamplifier with noise sources.

As shown in Figure 3, the sensor coil can be equivalent to a series circuit model composed of inductance *L* and resistance *r*, and a tuning capacitor *C* is connected in parallel to the sensor coil, which forms an LC resonant circuit, where *R* is the matching resistor that determines the *Q* of the circuit, and *G* is the gain of the preamplifier.

The thermal noise spectral density of the audio coil resistance contribution *e*_r_ is

(4) $$e_{r}=\sqrt{4kTr},$$

where *k* is the Boltzmann constant, and *T* is the temperature. The contribution of *e*_r_ to the noise at the preamplifier input node INP is

(5) $$e_{\mathrm{rINP}}^{2}=e_{r}^{2}/{\left(1+r/R-\omega^{2}/\omega_{0}^{2}\right)}^{2}+\omega^{2}{\left(Cr+L/R\right)}^{2},$$

where $\omega_{0}$ is the angular frequency of LC resonance and is

(6) $$\omega_{0}=1/\sqrt{LC}\;.$$

The thermal noise spectral density of the matching resistor contribution *e*_R_ is

(7) $$e_{R}=\sqrt{4kTR}\;.$$

The contribution of the matching resistor thermal noise *e*_R_ to the noise at the preamplifier input node INP is

(8) $$e_{\mathrm{RINP}}^{2}=e_{R}^{2}(r^{2}+\omega^{2}L^{2})/(R^{2}({\left(r/R+1\right)}^{2}+{\left(\omega /\omega_{0}\right)}^{4}+\omega^{2}\left(C^{2}r^{2}+L^{2}/R^{2}-2/\omega_{0}^{2}\right))).$$

The voltage noise of the preamplifier presents 1/*f* noise in the low-frequency band, and the noise corner frequency usually occurs at several Hz. The measurement range of the OVM is 851 Hz to 5109 Hz, so in this frequency band, the voltage noise *e*_n_ can be regarded as a constant, and its contribution to the noise of the preamplifier input node is also *e*_n_.

The current noise *i*_n_ of the preamplifier represents current fluctuations at the input of an otherwise noise-free amplifier with open inputs and can be expressed as

(9) $$e_{\mathrm{iINP}}^{2}=i_{n}^{2}\frac{\left(r^{2}+\omega^{2}L^{2}\right)}{\left((1+r/R-\omega^{2}/\omega_{0}^{2}{)}^{2}+\omega^{2}{\left(Cr+L/R\right)}^{2}\right)}.$$

The total noise at the preamplifier output is

(10) $$e_{\mathrm{op}}=G\sqrt{\left(e_{n}^{2}+e_{\mathrm{rINP}}^{2}+e_{\mathrm{RINP}}^{2}+e_{\mathrm{iINP}}^{2}\right)}.$$

Considering (4)–(10), $e_{op}^{2}$ is simplified as

(11) $$e_{\mathrm{op}}^{2}=G^{2}\left[e_{n}^{2}+\frac{e_{r}^{2}+\left(r^{2}+\omega^{2}L^{2}\right)\left(i_{n}^{2}+e_{R}^{2}/R^{2}\right)}{{\left(1-\omega^{2}/\omega_{0}^{2}\right)}^{2}+\omega^{2}C^{2}r^{2}}\right].$$

According to (11), we can see that the audio coil resistor thermal noise, the matching resistor thermal noise, and current noise are mainly affected by the resonant circuit, and their spectral densities reach a maximum at the resonance point and gradually decrease away from the resonance point. The voltage noise spectral density of the preamplifier, on the other hand, is not affected by the resonant circuit.

### 3.2. Impact of Transmission Characteristics on Noise

**Figure 4:**
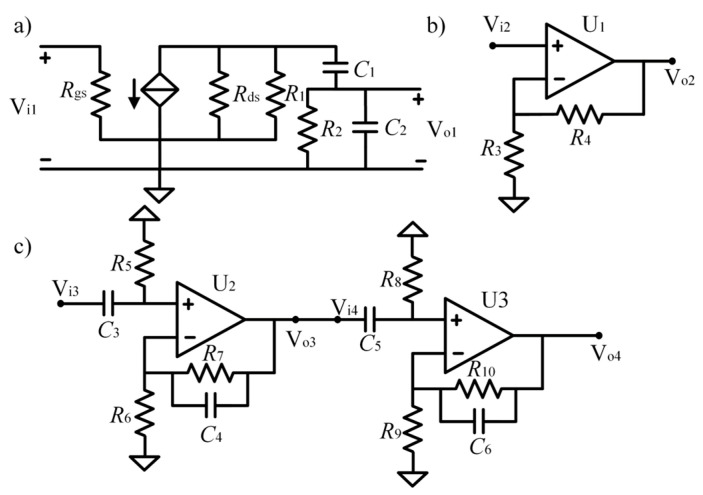
Signal conditioning circuits. (**a**) JFET preamplifier circuit small signal model. (**b**) Buffer amplifier circuit equivalent circuit. (**c**) Bandpass amplifier circuit equivalent circuit.

From the above analysis, it can be seen that the LC resonant circuit will affect the noise spectral density, and the signal conditioning circuit of the OVM is used to amplify and filter the signal, and its amplitude-frequency characteristics will also further affect the noise spectral density. In order to establish the comprehensive noise model of the OVM, the transmission characteristics of the signal conditioning circuit will be analyzed in this paper. The signal conditioning circuit of OVMs includes preamplification, buffer amplification, and bandpass filter amplification, as shown in Figure 4.

The preamplifier used is a low-noise JFET, the equivalent small signal model circuit diagram is shown in Figure 4a, and the transfer function is

(12) $$H_{1}=\frac{V_{o1}}{V_{i1}}=\frac{sC_{1}R_{2}R_{1}}{sC_{1}R_{2}+(1+sC_{1}R_{1})(sC_{2}R_{2}+1)}g_{m}.$$

The buffer amplifier circuit is used to increase the load capacity and reduce the influence of the load on the signal source. The equivalent circuit diagram is shown in Figure 4b, and its transfer function is

(13) $$H_{2}=\frac{V_{o2}}{V_{i2}}=1+\frac{R_{4}}{R_{3}}.$$

The bandpass filter amplifier is mainly designed to suppress low-frequency noise, such as 50 Hz, and to provide −40 dB/decade attenuation in the high-frequency section to ensure a stable amplitude of the FID signal within the measurement range. The equivalent circuit is shown in Figure 4c, and the first-stage transfer function is

(14) $$H_{3}=\frac{V_{o3}}{V_{i3}}=\frac{sR_{5}C_{3}}{sR_{5}C_{3}+1}(1+\frac{R_{7}}{R_{6}(1+sR_{7}C_{4})}).$$

Similarly, the transfer function of the second-stage circuit is

(15) $$H_{4}=\frac{V_{o4}}{V_{o3}}=\frac{sR_{8}C_{5}}{sR_{8}C_{5}+1}(1+\frac{R_{10}}{R_{9}(1+sR_{10}C_{6})}).$$

The transfer function *H*_s_ of the signal conditioning circuit is

(16) $$H_{s}=H_{1}\times H_{2}\times H_{3}\times H_{4}.$$

**Figure 5:**
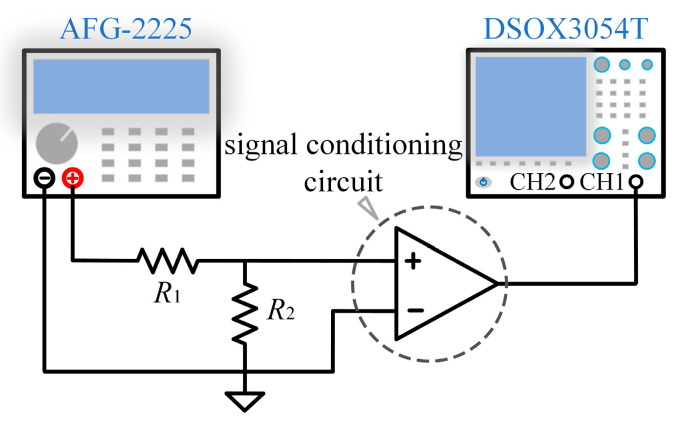
Signal conditioning circuit amplitude frequency response test schematic.

**Figure 6:**
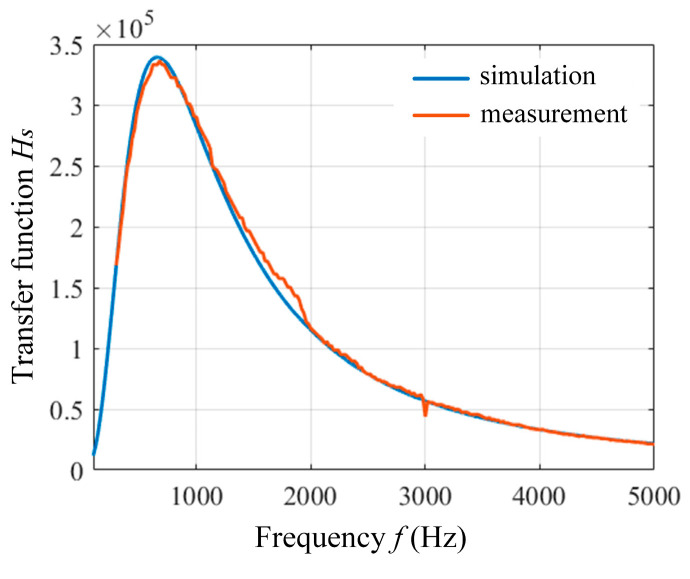
Signal conditioning circuit amplitude frequency response. Blue line: theoretical; orange line: measurement.

To verify the correctness of the theoretical model, we set up an experimental test platform in the laboratory, using a function generator (GW Instek AFG-2225, New Taipei City, Taiwan) to generate sinusoidal signals in the range of 500–5100 Hz and a high-precision oscilloscope (Keysight DSOX3054T, Santa Rosa, CA, USA) to measure the signals at the output, and the test configuration is shown in Figure 5. The measured transmission characteristic is shown in Figure 6 (orange curve). The theoretical calculation of the transfer function of the signal conditioning circuit was performed according to (12)–(16), and the results are shown in Figure 6 (blue curve). The results show that the theoretical calculation is highly consistent with the measured data, verifying the correctness of the transfer function model. According to the noise analysis results at the amplifier input node INP in the previous section, combined with the transmission characteristics of the signal conditioning circuit, we can establish the total noise model of the OVM, which can be expressed as

(17) $$e_{0}=H_{s}\times \sqrt{\left(e_{n}^{2}+e_{\mathrm{rINP}}^{2}+e_{\mathrm{RINP}}^{2}+e_{\mathrm{iINP}}^{2}\right)}+e_{e}.$$

where *e*_e_ represents the interference of EMI and other noise in the environment, which is random and uncontrollable, and depends only on the external environment.

## 4. Low-Noise OVM Design

As shown in (17), the output noise of the OVM mainly comes from resistive thermal noise, amplifier voltage noise, and amplifier current noise. In order to complete the design of a magnetometer with high SNR, this paper will further analyze the influence mechanism of each type of noise and discuss the strategy to reduce the output noise. When analyzing the impact of a certain type of noise on system performance, other noise sources will be assumed with reasonable reference values to ensure the pertinence of the analysis.

### 4.1. Matching Resistance Determination

The quality factor and bandwidth of the LC resonant circuit vary with the matching resistance *R*, and different *R* values correspond to different thermal noise. In order to determine the optimal *R*, an equivalent source impedance *R*_s_ at the input of the amplifier can be written as

(18) $$R_{s}=\frac{(r+j\omega L)R}{(r+j\omega L)(1+j\omega RC)+R}.$$

In order to optimize the selection of source impedance, we use the noise figure (*NF*) to estimate the effect of different source impedances on the noise performance of the system. The *NF* is defined as the ratio of the input *SNR*_in_ to the output *SNR*_out_, which can be expressed as

(19) $$NF=10\mathrm{lg}(SNR_{\mathrm{in}}/SNR_{\mathrm{out}})=10\mathrm{lg}(P_{o}/G^{2}P_{i}),$$

where *P*_o_ is the output noise power, which is composed of the current noise (*Gi*_n_*R*_s_)^2^, voltage noise (*Ge*_n_)^2,^ and source impedance thermal noise ($G\sqrt{4kTR_{s}}$)^2^; *P*_i_ is the noise power at the input, which is mainly contributed by the source impedance thermal noise. Supposing that the bandwidth of the LC resonant circuit is *BW*, after substituting each part of the noise, (19) can be further expressed as

(20) $$NF=10\mathrm{lg}(\frac{(e_{n}^{2}+i_{n}^{2}R_{s}^{2}+4KTR_{S})BW}{(4KTR_{S})BW})=10\mathrm{lg}(1+\frac{e_{n}^{2}+i_{n}^{2}R_{s}^{2}}{4KTR_{S}}).$$

**Figure 7:**
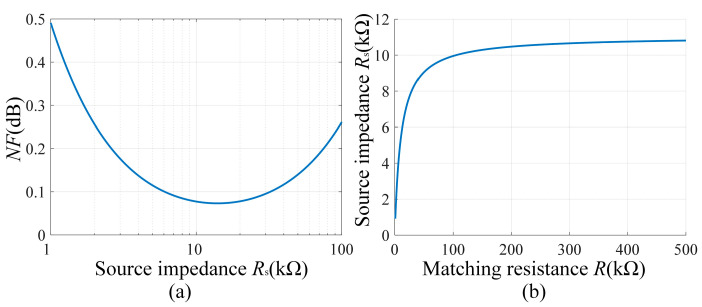
(**a**) The source impedance *R*_s_ vs. matching resistance *R*. (**b**) Noise figure *NF* vs. source impedance *R*_s_.

Suppose that the coil resistance, inductance, voltage noise, and current noise are 34.2 mH, 18.2 Ω, 1.4 nV/$\sqrt{Hz},$ and 0.1 pA/$\sqrt{Hz}$ [26], respectively, we calculate the effect of the source impedance *R*_s_ on the noise figure *NF* at 2.1 kHz according to (20), and the result is shown in Figure 7a. The results show that there exists a minimum value of the noise figure *NF* when the source impedance *R*_s_ is varied in the range of 1~100 kΩ, and the minimum value of *NF* occurs when *R*_s_ is between 10 kΩ and 20 kΩ.

In addition, we calculate the variation of source impedance *R*_s_ with matching resistance according to (19). It can be seen from Figure 7b that the source impedance *R*_s_ remains unchanged when the matching resistance is larger than 100 kΩ. Given that the minimum value of the *NF* corresponds to *R*_s_ of about 10~20 kΩ and considering that a larger matching resistance also means a higher quality factor *Q*, which helps to improve the resonance performance, the matching resistance is finally chosen to be 100 kΩ.

### 4.2. Parameter Analysis of Sensor Audio Coil and Preamplifier

The noise of the audio coil depends on its resistance, which is determined by both the number of turns and the wire diameter. When the number of turns is small and the wire diameter is large, the resistance is low, but the signal amplitude also decreases. To achieve a balance between signal strength and noise control, it is necessary to optimize the design of the coil, including the number of turns and wire diameter. For simplicity, we suppose that the wire diameter is constant and the influence of the number of turns on the SNR will be investigated to determine the optimal number of turns.

**Figure 8:**
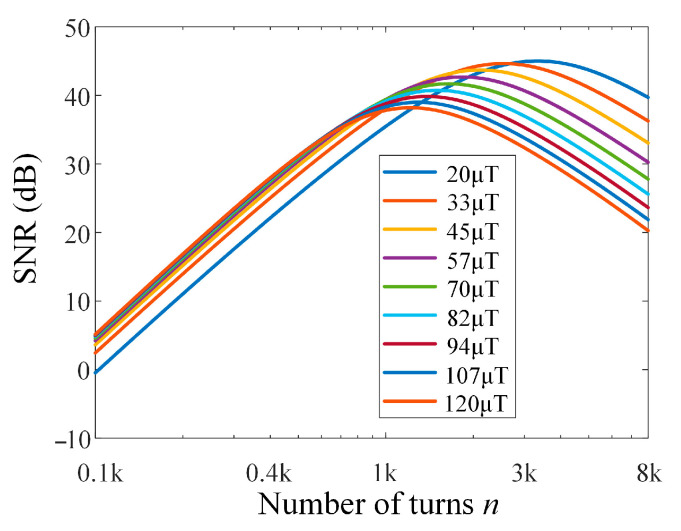
Number of turns *n* vs. SNR at different resonant frequencies.

The relationship between the resistance value and the number of turns of the coil is

(21) $$r=\frac{4n\rho d_{c}}{d^{2}},$$

where ρ is the coil resistivity, and *d* is the coil wire diameter. When the coil frame is determined, the inductance of the finite field multi-layer hollow coil is [27]

(22) $$L=k_{n}n^{2},$$

where *k*_n_ is the fixed coefficient. According to (3), the initial amplitude of the FID signal can be expressed by *n* as

(23) $$E_{s}=n\omega E_{0},$$

where *E*_0_ is the constant product in (3). The equivalent value of the noise at the output of the system can be calculated by the following equation

(24) $$E=\sqrt{\int_{f_{1}}^{f_{2}}e_{o}^{2}df},$$

where *f*_1_ and *f*_2_ are the lower and upper cutoff frequencies of the bandpass filter, respectively. The SNR of the OVM output is

(25) $$SNR=20\log\frac{QE_{S}H_{S}(f_{0})}{\sqrt{\int_{f_{1}}^{f_{2}}e_{0}^{2}df}},$$

where *f*_0_ is the LC circuit resonant frequency, and *H*_s_(*f*_0_) is the gain of the signal conditioning circuit transfer function *H*_s_ at the frequency *f*_0_. According to (17), (21)–(25), and assuming that the voltage noise of the preamplifier is 1 nV/$\sqrt{Hz}$ and the current noise is 0.1 pA/$\sqrt{Hz}$, the effect of the number of turns *n* on the SNR at different resonant frequencies is calculated, and the results are shown in Figure 8.

From the results in Figure 8, it can be seen that there is an optimal number of coil turns within the measurement range of 20 μT (851 Hz)–120 μT (5109 Hz). When the number of turns is around 1500, the SNR in the range of 20 μT–120 μT is averaged to the maximum value, and the difference in SNR between different field strengths is small. Insufficient turns result in a lower amplitude of the Larmor signal, leading to a poor overall SNR. However, excessive turns lead to a decrease in SNR and enlarge the difference in SNR. Moreover, increasing the number of turns increases the volume of the sensor, reducing the portability of the system. Comprehensively, we selected 1400 turns of enameled copper wire with a wire diameter of 0.54 mm, and the resistance and inductance are 22.5 Ω and 33.5 mH, respectively.

According to (9) and (24), we calculated the impact of the voltage and current noise of JFET on the root mean square (rms) value of the system output noise. The calculation results show that both the voltage noise and current noise output by the system increase linearly with the increase of JFET’s own noise, and this indicates that the noise performance of the device itself has a significant impact on the system output noise. In order to reduce system output noise, JFET devices with low voltage noise and low current noise should be prioritized, and these two parts of noise should not be higher than the coil resistance thermal noise. Based on this analysis, we select 2N6550 as the preamplifier, whose voltage noise is 1.4 nV/$\sqrt{Hz}$ and current noise is 0.1 pA/$\sqrt{Hz}$.

**Figure 9:**
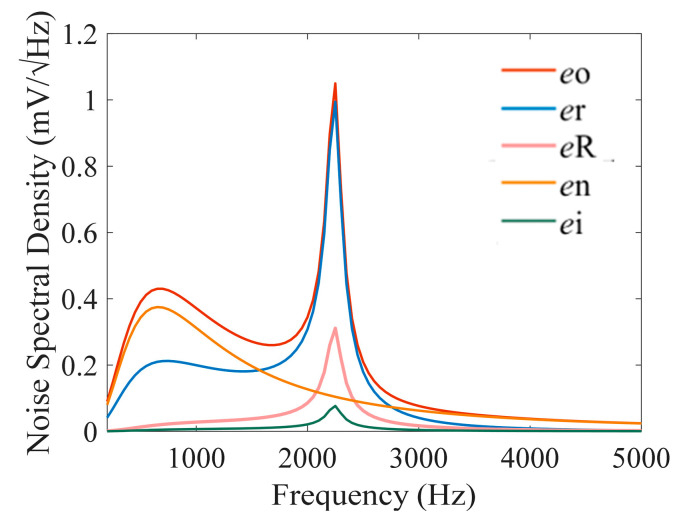
Estimated noise contributions after the signal conditioning circuit.

Finally, the optimized low-noise design parameters are listed in Table 1. Combining (17) and the parameters in Table 1, the calculated equivalent noise spectral density is shown in Figure 9. Results indicate that noise at resonant frequency is mainly contributed by the coil resistance thermal noise, whose noise density can reach 1.0 mV/$\sqrt{Hz}$; Outside the resonant frequency band, the main source of the noise is the voltage noise, whose maximum noise density can reach 0.4 mV/$\sqrt{Hz}$.

**Table 1:** Optimized vs. unoptimized audio coil and preamplifier electrical parameters.

Parameter	Optimized Value	Unoptimized Value
Audio coil resistance *r*	22.5 Ω	28 Ω
Audio coil inductance *L*	33.5 mH	34 mH
Matched resistance *R*	100 kΩ	50 kΩ
Resonance frequency *f*_0_	2.3 kHz	2.3 kHz
Gain of preamplifier *G*	32	32
Preamplifier voltage noise *e*_n_	$1.4\;\mathrm{nV}/\sqrt{Hz}$	$1.5\;\mathrm{nV}/\sqrt{Hz}$
Preamplifier current noise *i_n_*	$0.1\;\mathrm{pA}/\sqrt{Hz}$	$0.8\;\mathrm{pA}/\sqrt{Hz}$

According to (24) and the parameters in Table 1, the rms of different types of noise is calculated and shown in Table 2. The results show that the total noise output of the optimized system is 23.93 mV, compared to 28.6 mV for the unoptimized parameters, representing a theoretical noise reduction of 4.7 mV. The main noise sources are the thermal noise of the coil resistor (17.62 mV) and the voltage noise of the preamplifier (15.51 mV). Compared to the voltage noise and thermal noise of coil resistance, the contribution of the current noise and the thermal noise of the matching resistor is negligible.

## 5. Experimentation

### 5.1. Experimental Verification of Noise Model

**Figure 10:**
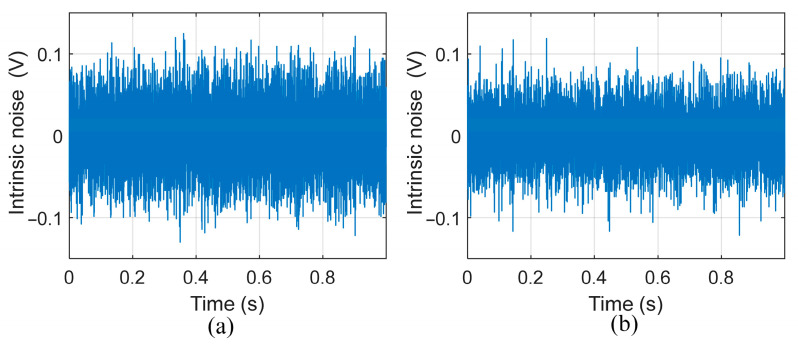
(**a**) Intrinsic noise in a strong EMI environment. (**b**) Intrinsic noise in a weak EMI environment.

In order to verify the correctness of the noise model proposed in this paper, we used a prototype OVM to measure the instrument’s intrinsic noise. The sensor was mounted on a 2 m high aluminum pole to avoid interference from ground surface magnetic materials and earth current fields. The intrinsic noise of the instrument was measured after the FID signal had completely attenuated. To observe the impact of environmental noise on the inherent noise, we used a handheld oscilloscope (ROHDE&SCHWARZ RTH1002, München, Germany) to record the intrinsic noise in the strong electromagnetic interference (EMI) environment on campus and the weak EMI environment in the countryside, and the results are shown in Figure 10.

According to the noise data recorded by the oscilloscope, the rms value of the noise *V*_nrms_ value under different EMI environments is calculated using (26). As shown in Table 3, it can be seen that the noise in the weak EMI environment is 26.7 mV, which coincides with the theoretically calculated value of 23.9 mV, verifying the accuracy of the theoretical values. The noise in the strong EMI environment is 32.3 mV, which is higher than that in the weak EMI environment, indicating the contribution of environmental noise.

(26) $$V_{\mathrm{nrms}}=\sqrt{\frac{1}{N}{\sum_{n=1}^{N}v_{n}}^{2}}.$$

**Figure 11:**
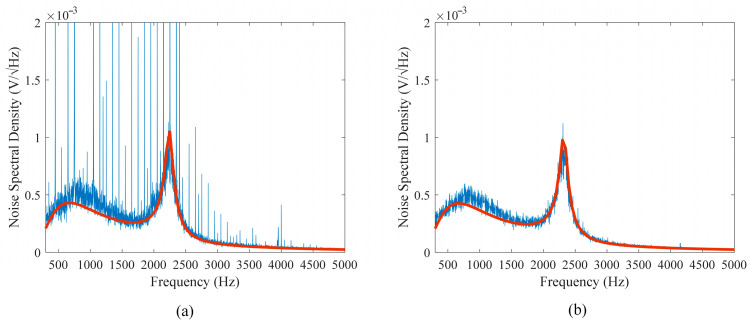
Noise spectrum of the instrument output. (**a**) Strong EMI environment. (**b**) Weak EMI environment. Blue line: measurement; orange line: theoretical.

Theoretical and experimental noise spectra density are also investigated in this paper. As shown in Figure 11a,b, the theoretical and experimental spectra density curves nearly coincide, confirming the validity of the proposed noise model. The LC resonance peak of the theoretical spectrum reaches approximately 1 mV/$\sqrt{Hz}$, while that of the measured spectrum is about 0.8 mV/$\sqrt{Hz}$. The slight difference between them is mainly due to the impedance of the matching capacitor (measured to be 68 kΩ at resonance), which is lower than the designed matching resistance of 100 kΩ, thereby reducing the effective *Q* of the resonant circuit.

The noise spectral density obtained under strong and weak EMI conditions reveals that, under strong EMI, multiple discrete spikes at 50 Hz and its harmonics indicate interference from external power lines. In contrast, under weak EMI conditions, there is no peak interference, indicating that these peaks come from environmental EMI rather than the inherent noise of the instrument.

### 5.2. Sensitivity Evaluated in Natural Magnetic Field

In this study, the sensitivity is quantitatively evaluated using the standard deviation (STD) of the raw data of the magnetic field being tested. To eliminate environmental interference and low-frequency interference, such as diurnal variation, data from two synchronized instruments in the natural magnetic field are usually subtracted from each other to obtain sensitivity. The calculated formula of sensitivity is [28]

(27) $$\sigma =\frac{1}{\sqrt{2}}\sqrt{\frac{1}{n-1}\sum_{i=1}^{n}{\left(B_{1i}-B_{2i}+{\bar{B}}_{2i}-{\bar{B}}_{1i}\right)}^{2}},$$

where *B*_1*i*_ and *B*_2*i*_ are the measurement values of the two instruments, $\bar{B_{1i}}$ and $\bar{B_{2i}}$ are the mean values of *B*_1*i*_ and *B*_2*i*_, and *n* is the number of measured samples.

**Figure 12:**
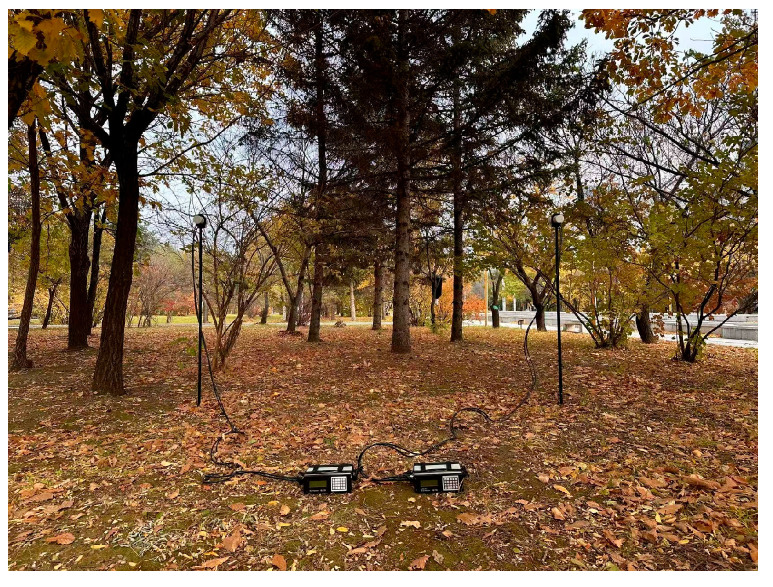
Natural magnetic field measurement.

As shown in Figure 12, the sensitivity of the magnetometer is estimated in the weak EMI environment. The clocks of the two magnetometers are synchronized, and the measurement cycle rate is set to 3 s. The sensors are mounted on a non-magnetic stand at a distance of 2 m to avoid interference.

**Figure 13:**
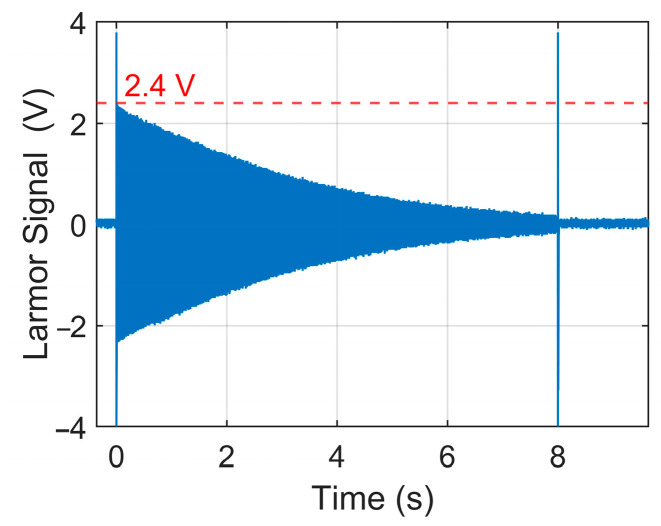
Measured Larmor signals.

The Larmor signal is measured, and the results are shown in Figure 13. The amplitude of the Larmor signal is 2.4 V, and the total noise of the system is 26 mV. The SNR can be calculated to be $10{log}_{10}{\left(2.4/0.026\right)}^{2}\approx 39.3$ dB. Compared with the unoptimized model, the SNR has been improved by 3 dB [29].

**Figure 14:**
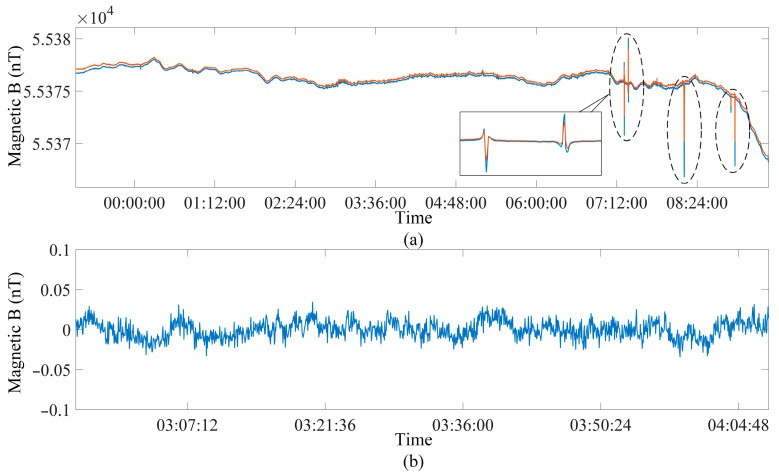
Natural magnetic field measurements. (**a**) Magnetic field values of the two synchronized instruments. Blue line: instrument #1; orange line: instrument #2. (**b**) Magnetic field difference at 3.00–4.00 a.m.

The magnetic field measurement results of two instruments are shown in Figure 14a, which indicates that the magnetic field measurements of the two instruments are highly consistent with the diurnal variation. The difference between the two instruments can be attributed to the gradient in spatial position. In the morning, significant fluctuations can be observed, which are caused by magnetic field anomalies triggered by passing vehicles. In order to minimize the influence of external noise and human activities on the sensitivity assessment, the sensitivity was evaluated at 1200 measurement points during the relatively quiet magnetic environment period from 3 a.m. to 4 a.m. The subtraction between the magnetic fields measured by the two magnetometers during this period is shown in Figure 14b. According to (27), the sensitivity is 0.0079 nT, which is 21% higher than the unoptimized design of 0.01 nT [29] without algorithm optimization. In addition, the sensitivity is also superior to the commercial instrument’s 0.01 nT [30].

In addition, the sensitivity was also evaluated in the frequency domain using the data collected during the quiet geomagnetic period from 3:00 a.m. to 4:00 a.m. The frequency-domain sensitivity was calculated based on the power spectral density (PSD) method, and the evaluation of the two synchronized instruments can be expressed as

(28) $$\mathrm{PSD}=\frac{1}{\sqrt{2}}\;|\;FFT(B_{1i}-B_{2i}+{\bar{B}}_{2i}-{\bar{B}}_{1i}),$$

**Figure 15:**
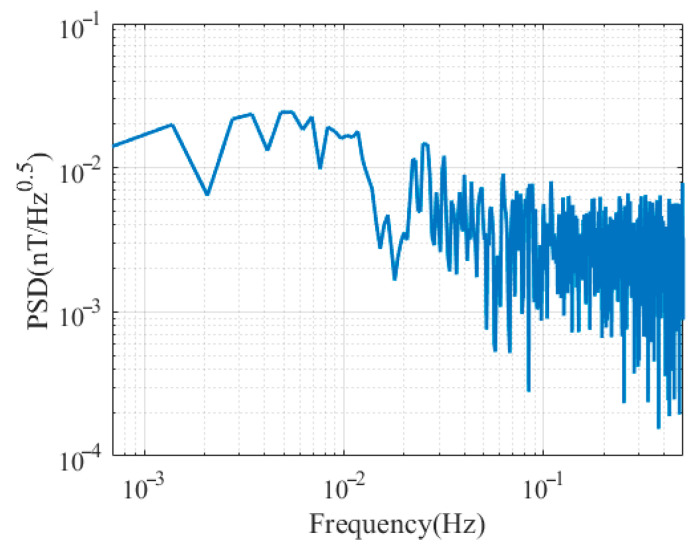
PSD results are between 3:00 a.m. and 4:00 a.m.

Figure 15 shows the frequency-domain sensitivity evaluation results. To eliminate the influence of geomagnetic diurnal variations and low-frequency 1/*f* system noise, the PSD stabilized curve is used to evaluate sensitivity. The results show that the frequency-domain sensitivity is approximately 0.008 nT/$\sqrt{Hz}$ in 0.1–0.5 Hz, consistent with the time-domain results.

## 6. Conclusions

An equivalent circuit model of noise is proposed to investigate the intrinsic noise characteristics of the OVM. Calculated results show that the intrinsic noise of the OVM mainly comes from the resistance thermal noise of the sensor audio coil and the voltage noise of the JFET preamplifier. The amplitude-frequency characteristics of the circuit significantly influence the noise bandwidth, thus affecting the final spectral characteristics of the noise.

The parameters of the OVM sensor, matching resistor, and preamplifier were optimized based on the noise model proposed in this article. The intrinsic noise of the system is measured in the natural environment, and the results show that the measured rms of the system noise is 26.7 mV, representing a 12% deviation from the theoretical value of 23.9 mV. Sensitivity is estimated in the natural magnetic field by using two synchronized instruments, and the sensitivity of the OVM is 0.0079 nT at 3 s. This sensitivity is significantly lower than the usual 0.01 nT at the same cycling time, indicating the effectiveness of this noise model in suppressing inherent noise. Compared to the method that improves SNR through signal processing algorithms, this method is simpler and more efficient, making it more suitable for real-time measurement.

The noise model proposed in this paper is not only suitable for low-noise OVM design, but also for other instruments with sensor coils.

## Figures and Tables

**Table 2:** Different noise output equivalents of OVMs.

Parameter	Value
Coil resistance thermal noise *e*_r_	17.62 mV
Matching resistance thermal noise *e*_R_	4.53 mV
Voltage noise *e*_n_	15.51 mV
Current noise *i*_n_	1.12 mV
Total noise *e*_o_	23.93 mV

**Table 3:** Different EMI environmental noise rms values.

	Theoretical Value	Strong EMI Environment	Weak EMI Environment
Noise rms	23.9 mV	32.3 mV	26.7 mV

## Data Availability

No new data were created or analyzed in this study. Data sharing is inapplicable to this article.
